# The International Tooth Crown Company *versus* the Dentists of the United States

**Published:** 1889-04

**Authors:** 


THE INTERNATIONAL TOOTH CROWN COMPANY VERSUS THE DENTISTS OF THE UNITED STATES.—What is the International Tooth Crown Company? What is it doing? It is a corporation not composed of dentists, but whose stockholders and active officers are parties recently connected with the Goodyear Dental Vulcanite Company, who for many years waged war on the individual dentists throughout the country; and its demands and *methods of operation* are very similar to those pursued by the old Goodyear Dental Vulcanite Company. There is this difference, however: the International Tooth Crown Company does not confine itself to any *one* patent, but is on the lookout for letters patent relating to dentistry when taken out by any person in the United States. It purchases such patents when practicable and then makes moneyed demands against dentists under them; and, if not submitted to, harasses them by suits in the United States Courts. It has recently, if we are correctly informed, added two new patents on removable bridges to its list.

These of the profession who consent to pay the royalty demanded are compelled to sign a license full of offensive agreements, and which is little less than a species of blackmail to say the least, or take the alternative of an expensive lawsuit.

The principal patents now owned by the International Tooth Crown Company are several patents known as tooth crown patents, which cover practically most of the successful operations in tooth crowning in use by our profession, and patents relating to bridge work, the principal one of these being the Low patent, under which they practically claim all bridge work.

The International Tooth Crown Company has suits now pending against members of our profession in the States of Maryland, New York, Connecticut and Wisconsin, and are about to commence suit in other States, or are threatening to do so.

A Connecticut and New York case was heard over two years ago, in which a voluminous record was made up on behalf of the complainants and defendants. Upon that record as made, the patent on bridge work was sustained, and an injunction issued against the defendant dentists. The bill, however, was dismissed as to the several patents on crown work, and an appeal was taken by the complainant, the International Tooth Crown Company, to the Supreme Court of the United States, where such patents are now pending.

To our personal knowledge the International Tooth Crown Company claims that the tooth crown patents will be unqualifiedly sustained in the coming trial before the Supreme Court of the United States, and that every member of our profession who has done tooth crown work will be subjected to litigation, put under injunction, and compelled to pay costs, damages and profits for infringement of these patents; and by reason of the voluminous and confused record on the part of the defendants in that case, there is great danger that this will be the result.

Our present annoyances from litigation are the claims of the Company on bridge work under the Low patent. The greatest danger, however, is that we will remain idle until the Supreme Court's decision on what are known as crown patents; then, if the decision is in favor of the Company's patents, as we anticipate, we will not be ready to meet them.

Now for the remedy. The only way to meet this problem is to get ready beforehand for the worst, viz.: the sustainment of these crown patents by the U. S. Supreme Court.

The profession should be in such shape that any member, however humble or obscure, or however remote from the large centers he may live, can step forward in any court, on short notice, with full and complete testimony to defeat these tooth crown patents, irrespective of and additional to any evidence presented in the case now pending in the U. S. Supreme Court.

To accomplish this single-handed and alone will involve the dentists in endless expense and trouble, and probably result in victory for the Company. *Concerted action is imperatively needed.*

With this end in view, at the earnest solicitation of many prominent members of the profession, "THE DENTAL PROTECTIVE ASSOCIATION OF THE UNITED STATES" has been formed. Its object is to contest, in a lawful and equitable manner, the patents of this Company or any other patents relating to dentistry, where the validity of such patent has not been fully established. After competent legal advice it was incorporated. This was accomplished under the name above set forth, and the undersigned consented to act as the first Board of Directors. The number of Directors was fixed at three, as it will be obvious to all that the work of such an organization to be prosecuted successfully must be in the hands of a small number, not widely separated, so that there may be concerted action without delay.

By-Laws have been adopted, a copy of which we will forward to you.

THE DENTAL PROTECTIVE ASSOCIATION OF THE UNITED STATES proposes to take charge of *all* suits commenced by the Company against any of its members *who unite themselves with this Association before such suits are commenced.*

And we state *unhesitatingly* and *advisedly* that with the evidence we now have in our possession and procurable, we will be able to defeat this Company in their suits against any member of the PROTECTIVE ASSOCIATION on a claim of either bridge or crown patents.

In order to do this we must have funds to engage the best legal talent, and to collect all the required evidence and place it in a form to be used in any part of the country. We therefore ask a general response from the profession. The membership fee is only $10.00, which will entitle the member to protection against this Company, or against any unjust prosecution on doubtful patents relating to dentistry.

The names of the members will not be made public.

We beg the hearty co-operation of every dentist in this movement. The benefits will be shared by all, while the work must mainly devolve upon a few. Dentists can greatly aid the Directors by *responding promptly*, and by inducing those whom this circular may not reach to join the Association. *Do not lay this upon the shelf until you have mailed your reply enclosing* $10.00.

We further desire any one having evidence, relating to either crown or bridge work, which evidence relates to crown and bridge work done before the year 1881, to send to us a detailed description of such work, enclosing a drawing and sketch of the work when completed, if possible, no matter how roughly such sketch may be made, and giving the name of the party for whom such work was made, and for how long a time used.

Please sign the enclosed By-Laws, and send name and address, *plainly written*, with membership fee, to J. N. CROUSE, Chairman, Board of Directors of the DENTAL PROTECTIVE ASSOCIATION, 2231 Prairie Avenue, Chicago, Ill., who will forward you a receipt.

LYMAN J. GAGE, Vice-President of the First National Bank of Chicago, has kindly consented to act as Treasurer for the Association.

J. N. CROUSE,<br>

T. W. BROPHY, } BOARD OF DIRECTORS.<br>

E. D. SWAIN,

LYMAN J. GAGE, TREASURER.

P. S.—To avoid unnecessary confusion and delay, *all communications should be sent to*

J. N. CROUSE, 2231 Prairie Avenue, Chicago, Ill.

CHICAGO, March 4, 1889.

